# First Report of *Campylobacter jejuni* Strains Belonging to ST-21 Clonal Complex Isolated from Human, Poultry and Wild Birds in Croatia: Antimicrobial Resistance and Genetic Distance

**DOI:** 10.3390/microorganisms11081884

**Published:** 2023-07-26

**Authors:** Silvija Šoprek, Josip Ujević, Gordan Kompes, Luka Jurinović, Arjana Tambić Andrašević

**Affiliations:** 1 Department of Clinical Microbiology, University Hospital for Infectious Diseases, 10000 Zagreb, Croatia; ssoprek@bfm.hr (S.Š.); jujevic@bfm.hr (J.U.); arjana.tambic@bfm.hr (A.T.A.); 2 Laboratory for General Bacteriology and Mycology, Department for Bacteriology and Parasitology, Croatian Veterinary Institute, 10000 Zagreb, Croatia; kompes@veinst.hr; 3 Laboratory for Bacteriology, Croatian Veterinary Institute, Poultry Centre, 10000 Zagreb, Croatia; 4 School of Dental Medicine, University of Zagreb, 10000 Zagreb, Croatia

**Keywords:** *C. jejuni*, ST-21 clonal complex, whole genome sequencing, cgMLST, antimicrobial resistance, host specialization, wild birds, human, poultry

## Abstract

In the era of growing antimicrobial resistance, a threat affecting humans, endangering animals, as well as livelihoods and food security worldwide, we wanted to find possible explanations for its continuous spread from a new perspective. The ubiquity of resistance genes requires a One Health approach to finding the explanations for continuous AMR spread. The natural transformability of *Campylobacter jejuni*, its high incidence of infections, and emerging resistance worldwide inspired us to choose *C. jejuni* ST-21CC to be our pathogen for analyzing its contribution and connection to the cycle of AMR dissemination. ST-21CC is known as a generalist among humans and broilers, the most prevalent lineage worldwide, but it is rarely found in wild birds. Emerging in wild birds, genetic relatedness and similar resistance profiles were expected. We analyzed 23 Croatian *C. jejuni* strains belonging specifically to ST-21CC from humans, broilers, and wild birds. The genomic data obtained through whole genome sequencing and phenotypic susceptibility data of strains were compared. Our findings suggest high fluoroquinolone resistance in ST-21CC strains, with more diverse genetic backgrounds in wild birds. Intriguing were three isolates of ST-822 (from human and storks), sharing a similar genetic fingerprint.

## 1. Introduction

Campylobacteriosis represents a global ongoing public health challenge since it is the leading zoonsis worldwide with the continuous increase in antimicrobial resistance of *Campylobacter* spp. [1,2,3,4,5,6,7,8]. Defined as a main cause of campylobacteriosis in both humans and animals, found emerging in wild animals, especially in wild birds, *C. jejuni* has proved to be an intriguing species for research. While genome analysis of *C. jejuni* strains from different sources enables the study of genetic variability and mutual relationships between them, defining the genetic determinants of antibiotic resistance of these bacteria can enable a better understanding of both the acquisition of resistance genes and their spread in the ecosystem. Everything said can lead to new insights into their pathogenesis and epidemiology which is of great importance for public health and food safety.

From an extremely rich pool of clonal complexes (CC) found in *C. jejuni* strains, ST-21 CC has been known as the largest and the most prevalent lineage among the human and broiler isolates worldwide. It also stands out for its distinct diversity compared to other clonal complexes; it is the most diverse CC in the *C. jejuni* population structure [9].

Generally, ST-21 CC is considered not to be a host- or niche-specific, also known as “specialist”, but rather a “generalist”, meaning that it can colonize a wide variety of hosts. Despite their ability to colonize a wide variety of hosts, including not only the digestive tract of birds (especially broilers), cattle, sheep, and pigs, but also wild animals and the environment (e.g., soil and water), strains of *C. jejuni* belonging to ST-21 CC were just recently observed emerging in wild birds in Croatia, found for the first time in Yellow-legged gulls and storks (present study).

The aim of the study was to investigate all ST-21 CC strains of *C. jejuni* obtained from several niches, including human, broiler, and wild birds, identified in our prior studies [10], as we found this ST-21 CC emerging in wild birds. As it was observed that ST-21 CC carries high resistance to ciprofloxacin and tetracycline, it poses a danger for further spread in the ecosystem. Sequentially, we designed this study to use the One Health approach as we found it could unveil some answers in the relation between campylobacter human infections and wild birds’ carriage as the One Health approach is a holistic and interdisciplinary strategy that aims to address health challenges at the interface of humans, animals, and the environment, considering their shared risks, promoting proactive measures, and enhancing the overall well-being of all. By recognizing the interdependencies between these domains, this approach offers a comprehensive framework for addressing emerging infectious diseases, antimicrobial resistance, zoonotic diseases, and environmental health issues effectively.

Phenotypic resistance, together with its underlying genetic determinants, is analyzed, with special focus put on observing the possible correlations between phylogenetic linkage of the three reservoirs.

## 2. Materials and Methods

### 2.1. Preparing the Bacterial Isolates

#### 2.1.1. Campylobacter Isolates

In this study, 23 Croatian *C. jejuni* strains belonging specifically to ST-21 CC found in a human, broilers, and wild birds (gulls and storks) were analyzed. The sample consisted of 8 human strains, 5 strains from broilers, 2 strains found in gulls and 8 strains from storks.

The majority of the strains were selected from the collection of *C. jejuni* at the Croatian Veterinary Institute, collected within other studies on *Campylobacter* spp. during a period of 2021–2022 [10].

The human strains were collected in the University Hospital for Infectious Diseases (samples ZG01, ZG02, ZG03, ZG06, ZG07, ZGI07, ZG12) and included strains from stool and blood samples of patients with diarrhea also during 2021 and 2022. These isolates were previously analyzed for the antimicrobial resistance [10] and added to other ST-21 isolates.

The strains originating from gulls were isolated from breeding Yellow-legged Gulls, Larus michahellis, in the Croatian part of the Adriatic Sea. Namely, 1-C-258 was isolated from an adult bird breeding on a rooftop colony in the city of Zadar and 1-C-381 was obtained from pullus sampled on the islet of Mrkan, near Dubrovnik. Most of the stork isolates are from chicks of White Storks, Ciconia ciconia, hatched in the Lonjsko Polje Nature Park (1-C-402, 1-C-405 and 1-C-406 from village Jasenovac; 1-C-410, 1-C-411 and 1-C-412 from village Puska), while 1-C-423 was isolated from a storklet from Jakuševec, Zagreb, breeding just near the rubbish tip.

The broiler isolates were taken from the Croatian Veterinary Institute collection of *C. jejuni* strains found in *Gallus gallus* chicks bred for meat production while implementing national monitoring programs for *Campylobacter* spp. in broilers. Everything was performed according to the amendment of Regulation (EC) 2073/2005 to include *Campylobacter* process hygiene criterion [11]; regulation (EC) No 2160/2003 [12].

Once collected in the laboratory of the Croatian Veterinary Institute, strains were streaked on blood agar supplemented with 10% of defibrinated sheep blood (Columbia agar, Biomerieux, Marcy-l’Étoile, France) and incubated in microaerobic conditions (CampyGen, Thermo Fischer Scientific, Oxoid Limited, Hants, UK) at 42 °C for 48 h. Once revived, they were streaked again (sub-cultured) on blood agar supplemented with 10% of defibrinated sheep blood and stored at −80 °C for all necessary additional analyzes if needed. All the strains were studied and analyzed in the collaboration of the Croatian Veterinary Institute and the University Hospital for Infectious Diseases laboratories.

#### 2.1.2. Species Confirmation

All strains were further subjected to species confirmation performed by a multiplex PCR assay according to Wang et al., 2002 [13]. This method provides the possibility of detecting genes from the five major clinically relevant *Campylobacter* species simultaneously. For species confirmation, DNA extraction was performed on fresh, overnight culture of *C. jejuni* strains. Strains were streaked and cultured on blood agar supplemented with 10% of defibrinated sheep blood and incubated in microaerobic conditions overnight when reviving bacteria. A full loop of pure culture was taken and resuspended in 100 μL of PCR clean water. DNA was extracted from the prepared suspension using the NucleoSpin Microbial DNA Mini Kit (Macherey-Nagel, Düren, Germany) according to the manufacturer’s instructions. DNA samples were sent to MicrobesNG LTD for whole genome sequencing (WGS).

#### 2.1.3. Antimicrobial Susceptibility Testing (AST)

Strains were revived and sub-cultured on blood agar supplemented with 10% defibrinated sheep blood. Following overnight incubation, the cultures of *C. jejuni* strains were used for antimicrobial susceptibility testing (AST). Using the broth microdilution method, AST was carried out for six antimicrobials of clinical and epidemiological interest, following the European Committee on Antimicrobial Susceptibility Testing (EUCAST) guidelines. For this testing, EUCAMP3 microplates were used (Sensititer, Trek Diagnostic Systems Ltd. East Grinstead, West Sussex, UK). Epidemiological cut-off values (ECOFFs) were used for interpretative thresholds for resistance, identifying the non-wild-type strains defined as potentially harboring resistance mechanisms. EUCAST ECOFFs were used to determine susceptibility to erythromycin (ERY; 1–512 mg/L), ciprofloxacin (CIP; 0.12–32 mg/L) and tetracycline (TET; 0.5–64 mg/L), while for ertapenem (ERTA 0.12–4 mg/L), gentamicin (GEN; 0.25–16 mg/L), and chloramphenicol (2–64 mg/L), EFSA ECOFFs were used, as for these antibiotics there are no available data by the EUCAST [14,15]. For quality control of susceptibility, to ensure that the results were within acceptable limits, the *C. jejuni* ATCC 33,560 reference strain was used.

### 2.2. Whole Genome Sequencing

#### 2.2.1. Genomics

Genomic DNA samples extracted from 23 *C. jejuni* isolates were sent to MicrobesNG (Birmingham, UK) for sequencing. The Illumina platform (Illumina, Inc., San Diego, CA, USA) was used for the 2 × 250 bp paired-end sequencing of genomic libraries. The results were obtained as raw trimmed reads and assembled FASTA files.

The basic bioinformatic analysis was also provided by MicrobesNG (Birmingham, UK) through their standard pipeline. The closest available reference genome was identified using the Kraken version 1.1 software, and the reads were mapped to this using the BWA mem tool version 0.7.11 (Burrows–Wheeler Aligner) to assess the quality of the data. A de novo assembly of the reads was performed using SPAdes version 3.15.5, and the reads mapped back to the resultant contigs, again using the BWA mem to obtain more quality metrics.

#### 2.2.2. Genetic Distance Analysis

Multi-locus sequence typing (MLST) analysis was performed on de novo assemblies to genetically characterize *C. jejuni* isolates. As a result, sequence types (ST) and clonal complexes (CC) were determined using the publicly available pubMLST database, (http://pubmlst.org/campylobacter, accessed on 15 December 2022).

**Figure 1:**
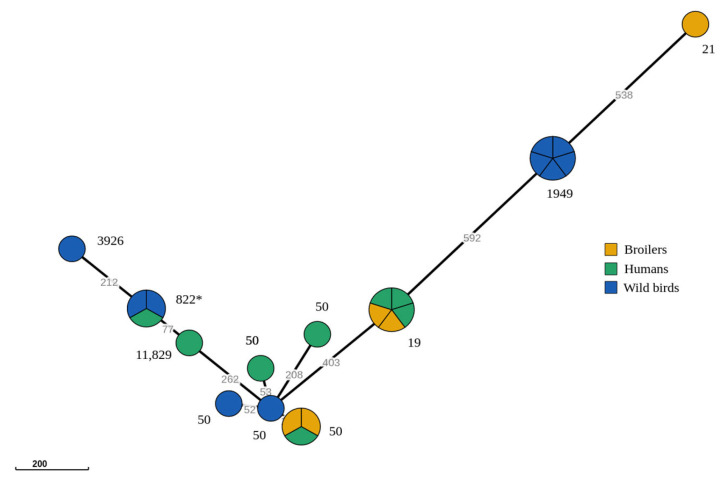
Minimum spanning tree (MST) revealing genomic relationship amongst *C. jejuni* ST-21 CC based on cgMLST profiles. Different colors represent different reservoirs of strains. The numbers next to the samples correlate with the identified STs. The asterisk mark indicates clusters that are phenotypically defined as fluoroquinolones wild-type strains.

Additionally, to extend the concept of MLST to a larger number of genes presented in the genome, an analysis of the core genome MLST (cgMLST) was performed using the publicly available cgMLSTfinder (https://cge.food.dtu.dk/services/cgMLSTFinder/, accessed on 15 December 2022). The cgMLST loci scheme (*n* = 1343) [16] was retrieved from the *Campylobacter* PubMLST database [9]. Raw reads in a fastq format were used as input data. A minimum spanning tree was created using the Grapetree version 1.5.0 software to evaluate the relatedness amongst *C. jejuni* isolates based on cgMLST allele profile (Figure 1).

#### 2.2.3. Identification of Antibiotic Resistance Genes

In silico AST analysis was performed on de novo assemblies using the publicly available CARD’s Resistance Gene Identifier (RGI) bioinformatic tool (https://card.mcmaster.ca/home, accessed on 16 December 2022) and ResFinder 4.1 (https://cge.food.dtu.dk/services/ResFinder, accessed on 16 December 2022). For both packages, default parameter values were used to identify resistance determinants. All 23 *C. jejuni* genomes were analyzed for the acquired antimicrobial resistance genes and chromosomal point mutations with a threshold of 98% for ID and 100% for minimum length.

## 3. Results

### 3.1. PCR Identification and Genetic Distance Analysis

All the 23 tested strains were identified as *C. jejuni* using PCR according to Wang et al. 2002. WGS data were used to determine sequence types (ST), clonal complex (CC), core genome sequence type (cgST) and to identify resistance determinants for strains included in this study. The main goal of this study was to analyze isolates belonging to the ST-21 CC, specifically to determine genetic diversity and the genotypic–phenotypic correlation of the isolates from this CC.

The most prevalent ST in examined isolates was 50 (*n* = 6), followed by 19 (*n* = 5) and 1949 (*n* = 5). To gain greater insight into the genetic background of the analyzed strains, cgMLST analysis was performed. Results of this type of analysis revealed that the group of isolates belonging to ST-50 can be further divided into three subgroups based on the examined allele profiles: three isolates belong to the cgST-294 subgroup, two isolates belong to the cgST-35358 subgroup and one isolate belongs to the cgST-10207 subgroup. The ST-19 cluster of *C. jejuni* was detected in three human samples and in two samples from broilers, and all the detected isolates in that cluster correlate with the same cgST-2499. A similar pattern shows the ST-1949 group of isolates, which were detected only in samples from white storks, and they all correlate with cgST-7821. A list of all ST, cgST, specimen and source of isolation can be found in Table 1.

### 3.2. Genomic Relationship amongst C. jejuni ST-21 CC Strains

Data obtained from cgMLST analysis was used to visualize the genomic relationship amongst the isolates belonging to ST-21 CC. Minimum spanning tree was created for all 23 isolates using the cgMLST *C. jejuni* scheme (Figure 1).

For 14 isolates (61%), alleles were assigned to more than 90% of all cgMLST loci. For eight isolates (35%), alleles were assigned in the range from 80% to 90% of loci from the *C. jejuni* scheme. For the remaining isolate, the percentage of assigned alleles was 76.62. On average, 89.81% of alleles were assigned.

The presence of 10 lineages that correlate with cgMLST profiles are revealed with this analysis. There are four different clusters, with a maximum of five and minimum of three isolates, and seven singletons. The most abundant clusters are STs 19 and 1949, as they have total number of five isolates per cluster. Cluster ST-1949 consists of strains isolated only from wild birds, whereas the ST 19 group comprises the strains isolated from three human and two boiler samples. Based on cgMLST allele profile analysis, ST 50 group shows more dispersive profiles, forming one cluster and three singletons. The ST 50 cluster consists of three isolates which were isolated from two broiler samples and from one human sample, as shown in Figure 1.

### 3.3. Antibiotic Resistance Phenotypes

Among all the 23 strains tested, the phenotypic resistance was found only in the antibiotic class of fluoroquinolones. In vitro susceptibility testing shows that even 83% of the isolates (19 of 23 isolates) exhibit MIC values of ciprofloxacin higher than those of the ECOFFs, meaning that 83% of the isolates included in this study are defined as non-wild-type isolates (NW). The isolates exhibiting MIC values of ciprofloxacin lower than those of ECOFFs, meaning they were defined as wild-type isolates (W), specifically belong to ST 822. One of them was a human strain while the other two strains were found in storks. Results from erythromycin, ertapenem, gentamicin, tetracycline, and chloramphenicol testing show that all tested strains were classified as wild-type strains. Results of AST regarding six antimicrobial agents determined by the broth microdilution method are presented in Table 2.

### 3.4. Identification of Antibiotic Resistance Genes and Mutations

Resistance to ciprofloxacin (fluoroquinolones) was genotypically the most represented and extremely high with mutation in *gyr*A detected in 18 out of the 23 tested strains (78%). All the tested strains have the same T86I mutation. It is a point mutation resulting in a substitution of ACA with ATA in the quinolone resistance-determining region (QRDR) of the *gyr*A gene.

Four strains without a detected *gyr*A mutation clustered specifically to the cgST-115 clone, found in two stork strains and two human strains.

Genes connected to a possible beta-lactam resistance (*bla*OXA genes) were identified in 22 out of the 23 strains tested (96%). All the identified *bla*OXA genes belong to class D β-lactamase OXA-61, with only two variants of genes present in the tested strains, OXA-193 and OXA-580, respectively. There is an evident predominance of the OXA-193 variant as it was detected in 19 isolates, while the OXA-580 variant was found in only five isolates. Not a single genetic determinant of resistance was found responsible for carbapenem, tetracycline, aminoglycoside, macrolides nor chloramphenicol resistance in any of the tested strains.

### 3.5. Correlations between Phenotypic and Genotypic Resistance

Overall, the phenotypic and genotypic antimicrobial predictions were absolutely correlated without any discordances between phenotypic and genotypic results.

Mutations in *gyr*A resulting in reduced susceptibility to ciprofloxacin were detected in 18 of 23 isolates. Five out of six antimicrobial agents including erythromycin, gentamicin, chloramphenicol, and ertapenem resulted in both genotypic and phenotypic susceptibility (100%). The *bla*OXA gene was found in almost all our strains (in 22 out of 23), all belonging to a class D β-lactamase called OXA-61, with two variants present, OXA-193 and OXA-580. None of those variants, as far as we know, should confer resistance to carbapenems [17,18]. The only strain lacking the *bla*OXA gene was ZG07, a human strain belonging to ST 2787. There were no major errors nor very major errors detected in the results.

The correlation between the results of antimicrobial susceptibility testing to six antimicrobial agents and WGS-derived antimicrobial resistance is presented in Table 3.

## 4. Discussion

The *Campylobacter jejuni* clonal complex ST-21, a generalist among humans and broilers, is recognized as the most prevalent lineage worldwide, but nevertheless it is rarely found in wild birds [9]. The occurrence of this clone in wild animal populations in Croatia, in our case, specifically in wild birds, seemed significant from several perspectives.

Namely, the problem of AMR is not only a human health-related issue but rather a globally growing threat affecting humans, endangering animals as well as livelihoods and food security worldwide [19,20,21]. The usage of antibiotics in both human and animal treatment, along with the presence of resistant bacteria, can lead to their dissemination through wastewater [19]. As a result, the bacteria may spread into the soil, contaminate waterways, and subsequently impact various forms of life, including wildlife. Consequently, the exchange of resistance genes and antibiotic-resistant bacteria spreads across borders and sectors, and unfortunately is not limited to some bacterial species. This ubiquity of resistance genes requires a One Health approach in finding the explanations and underlying mechanisms for AMR spread and taking steps in the right direction.

Generally, it can be said that the presence of AMR found in wildlife is directly related to human activity and pressure consequently put on ecosystems, so some animals unfortunately become sentinels for AMR spread in the environment. Seagulls are an especially good example for AMR studies in bird species. Because they can travel long distance in a short period of time, it is supposed they can easily (being in contact with different regions with variable prevalence of AMR) acquire resistance genes both from the environment and the resistant bacteria while inhabiting high-risk regions.

The other issue is enthronization of ecosystems which has forced animal species to change their ecology, coexisting today as urban wildlife adapting to human presence. In that sense, some birds that were considered to be migratory tend to spend winters in Europe (for example, white storks—*Ciconia ciconia*, pers. data); consequently, more resistant bacteria have already been described in storks [22]. Considering everything mentioned above, our initial hypothesis of the study was that the selected isolates would show both genetic relatedness and share similar resistance profiles, both proven in our study.

In our study, just two AMR genes were identified, including point mutation in *gyr*A and the gene for the D class of beta lactamase belonging to the OXA-61 class, including the OXA-193 and OXA-580 variants. OXA-61, together with the point mutation in *gyr*A, were the most prevalent AMR genes in our strains. Identified OXA-61 has been shown to be identical to other OXA-type enzymes found in *Pseudomonas aeruginosa*, *Acinetobacter baumannii*, and Fusobacterium, conferring resistance to narrow-spectrum beta lactams (penicillin, oxacillin, ampicillin, amoxicillin-clavulanate, piperacillin, and carbenicillin) [17,18], so the analyzed strains did not show phenotypical resistances to ertapenem (carbapenems).

The findings of this study are in line with those of a previous study [23], thereby suggesting high resistance rates to fluoroquinolones in ST-21 CC. All mutations found in *gyr*A had the same point mutation T86I, including the nucleotide substitution of ACA to ATA. This was found to be the most prevalent mutation globally as well, especially linked to strains found in humans and poultry [23,24]. Previous studies also associate ST-21 CC with high resistance to tetracycline, but in our study, this was not the case [25].

Higher discriminatory power of cgMLST [26] was used to cluster ST-21 CC isolates of *C. jejuni*. This analysis revealed that the group of the examined strain forms a diverse and distinctive cluster. When observing the clustering of cgMLST, we noticed that the ST 50 group, which is also generally the most prevalent worldwide [9], does not form one cluster, but instead forms multiple clusters based on cgMLST profiles. In this study, we found that strains isolated from wild birds and broilers do not cluster together.

When focusing on fluoroquinolone-resistant strains of *C. jejuni* from our study, it is evident that the strains from humans and broilers share a certain genetic similarity, while the isolates found in wild birds tend to have a more diverse genetic background.

As mentioned above, given the increasingly frequent and close relationship of wild birds and humans and broilers nowadays, the initial hypothesis was proven in our study as all the examined isolates shared phenotypically similar patterns, suggesting only high resistance rates to fluoroquinolones in ST-21 CC, while genetic relatedness was found only between wild birds and human isolates, with sporadic overlaps detected in *C. jejuni* isolates found in humans and storks. Although colonization and infections of storks with more resistant types of bacteria have already been described [22], we did not observe this phenomenon to be connected with the population of *C. jejuni* analyzed in our study.

## Figures and Tables

**Table 1:** General information about tested strains.

Isolate ID	Source	Year of Isolation	Specimen	MLST	cgMLST
ZG01	human	2021	stool	19	cgST-2499
ZG02	human	2021	stool	19	cgST-2499
ZG03	human	2021	stool	50	cgST-10207
ZG04	human	2021	stool	19	cgST-2499
ZG06	human	2021	stool	822	cgST-115
ZG07	human	2021	stool	2787	cgST-35061
ZGI07	human	2021	blood	11,829	cgST-115
ZG12	human	2021	stool	50	cgST-294
1c258	*Larus michahellis*, Yellow-legged Gull	2021	cloacal swab	50	cgST-35358
1c381	*Larus michahellis*, Yellow-legged Gull	2022	cloacal swab	3926	cgST-27074
1c402	*Ciconia ciconia*, White Stork	2022	cloacal swab	822	cgST-115
1c404	*Ciconia ciconia*, White Stork	2022	cloacal swab	1949	cgST-7821
1c405	*Ciconia ciconia*, White Stork	2022	cloacal swab	ST-50	cgST-35358
1c406	*Ciconia ciconia*, White Stork	2022	cloacal swab	1949	cgST-7821
1c410	*Ciconia ciconia*, White Stork	2022	cloacal swab	1949	cgST-7821
1c411	*Ciconia ciconia*, White Stork	2022	cloacal swab	1949	cgST-7821
1c412	*Ciconia ciconia*, White Stork	2022	cloacal swab	1949	cgST-7821
1c423	*Ciconia ciconia*, White Stork	2022	cloacal swab	822	cgST-115
CP01	broiler	2021	cloacal swab	19	cgST-2499
CP03	broiler	2021	cloacal swab	19	cgST-2499
CP10	broiler	2021	cloacal swab	21	cgST-36048
CP11	broiler	2021	cloacal swab	50	cgST-294
CP14	broiler	2021	cloacal swab	50	cgST-294

**Table 2:** Distributions of MICs values among tested strains.

Antimicrobial Agent (Class)—MIC						
Isolate ID	Erythromycin (Macrolide)	Ciprofloxacin (Fluoroquinolones)	Tetracycline (Tetracycline)	Gentamicin (Aminoglycoside)	Chloramphenicol (Amphenicol)	Ertapenem (Carbapenem)
ZG01	≤1	16	≤0.5	≤0.25	4	≤0.12
ZG02	≤1	8	≤0.5	0.5	≤2	0.25
ZG03	≤1	8	≤0.5	0.5	≤2	0.5
ZG04	≤1	16	≤0.5	0.5	≤2	≤0.12
ZG06	≤1	≤0.12	≤0.5	0.5	≤2	≤0.12
ZG07	≤1	8	≤0.5	≤0.25	≤2	≤0.12
ZGI07	≤1	≤0.12	≤0.5	0.5	≤2	≤0.12
ZG12	≤1	8	≤0.5	0.5	≤2	0.5
1c258	≤1	0.12	≤0.5	0.5	≤2	0.25
1c381	≤1	8	≤0.5	0.5	≤2	≤0.12
1c402	4	≤0.12	≤0.5	0.5	≤2	≤0.12
1c404	≤1	8	≤0.5	≤0.25	≤2	≤0.12
1c405	≤1	8	≤0.5	0.5	≤2	≤0.12
1c406	≤1	8	≤0.5	≤0.25	≤2	≤0.12
1c410	≤1	8	≤0.5	0.5	≤2	≤0.12
1c411	≤1	8	≤0.5	≤0.25	≤2	0.25
1c412	≤1	8	≤0.5	≤0.25	≤2	0.25
1c423	≤1	≤0.12	≤0.5	0.5	≤2	≤0.12
CP01	≤1	16	≤0.5	0.5	4	0.25
CP03	4	8	≤0.5	≤0.25	≤2	≤0.12
CP10	≤1	16	≤0.5	0.5	4	0.25
CP11	≤1	8	≤0.5	≤0.25	≤2	0.25
CP14	≤1	8	≤0.5	≤0.25	≤2	0.25

**Table 3:** Comparison of phenotypic and genotypic results of tested strains (RD—resistance determinants, W—wildtype, NW—non-wild-type).

Isolate ID	Antimicrobial Agents (Class) and Resistance Determinants			
	Ciprofloxacin (Fluoroquinolones)	RD	Ertapenem (Carbapenem)	RD
ZG01	NW	*gyr*A T86I	W	OXA-580
ZG02	NW	*gyr*A T86I	W	OXA-193
ZG03	NW	*gyr*A T86I	W	OXA-193
ZG04	NW	*gyr*A T86I	W	OXA-193
ZG06	W	-	W	OXA-193
ZG07	NW	*gyr*A T86I	W	OXA-580
ZGI07	W	-	W	OXA-193
ZG12	NW	*gyr*A T86I	W	-
1c258	W	-	W	OXA-193
1c381	NW	*gyr*A T86I	W	OXA-580
1c402	W	-	W	OXA-580
1c404	NW	*gyr*A T86I	W	OXA-193
1c405	NW	*gyr*A T86I	W	OXA-193
1c406	NW	*gyr*A T86I	W	OXA-193
1c410	NW	*gyr*A T86I	W	OXA-193
1c411	NW	*gyr*A T86I	W	OXA-193
1c412	NW	*gyr*A T86I	W	OXA-193
1c423	W	-	W	OXA-580
CP01	NW	*gyr*A T86I	W	OXA-193
CP03	NW	*gyr*A T86I	W	OXA-193
CP10	NW	*gyr*A T86I	W	OXA-193
CP11	NW	*gyr*A T86I	W	OXA-193
CP14	NW	*gyr*A T86I	W	OXA-193

## Data Availability

Data available on request.
